# New Trends and Evidence for the Management of Renal Angiomyolipoma: A Comprehensive Narrative Review of the Literature

**DOI:** 10.15586/jkcvhl.v9i1.177

**Published:** 2022-01-21

**Authors:** Juan Camilo Álvarez Restrepo, David Andres Castañeda Millan, Carlos Andres Riveros Sabogal, Andres Felipe Puentes Bernal, Wilfredo Donoso Donoso

**Affiliations:** 1 Department of Urology, School of Medicine, Universidad Nacional de Colombia, Bogotá, Colombia;; 2 Urology Research and Innovation Group, Universidad Nacional de Colombia, Bogotá, Colombia;; 3 School of Medicine, Universidad Nacional de Colombia, Bogotá, Colombia;; 4 Department of Urology, Hospital Universitario Nacional de Colombia, Bogotaá, Colombia

**Keywords:** angiomyolipoma, diagnosis, kidney neoplasms, review, therapeutics

## Abstract

Treatment of renal angiomyolipoma (AML) seeks to reduce related complications and preserve kidney function. The purpose of this article was to perform an updated literature review on the diagnosis, therapeutic options, and criteria for invasive intervention in patients with renal AML. Computerized tomography is the standard diagnostic method for renal AML, while definitive diagnosis is made by histopathology. The management of choice in most cases is active surveillance (AS), with a clinical and imaging follow-up protocol. In high-risk cases, therapeutic management should be considered, with alternatives such as selective arterial embolization (SAE), nephron-sparing surgery (NSS), and mTOR inhibitors in selected patients. Renal AML in women of childbearing age, those with growth >0.25 cm/year, intralesional aneurysms >5 mm, and clinically significant symptoms may qualify for active treatment. Despite the limitations derived from the available evidence, it is possible to consider SAE, NSS, and the use of mTOR inhibitors as management alternatives for selected patients.

## Introduction

Renal angiomyolipoma (AML) is a rare benign renal tumor that is part of the group of perivascular epithelioid cell neoplasms (PEComas) (1, 2). It is derived from mesenchyme, and is thus composed of mature adipose tissue, smooth muscle, and dysmorphic blood vessels (3). Immunohistochemistry tends to be positive for the expression of HMB-45, melan-A, actin, desmin, and calponin (4).

It is responsible for 1–3% of kidney tumors with an incidence of 0.3–3% in the general population; it has a female-to-male ratio of 2:1 (2). Fifty to seventy percent of cases correspond to sporadic renal AML, characterized by a smaller size (average 1–4 cm), slow growth (0.19 cm/year), unilateral presentation, and an average age between 43 and 53 years at diagnosis (3, 5–7). The remaining 30–50% is associated with genetic syndromes such as sporadic lymphangioleiomyomatosis (LAM) and tuberous sclerosis complex (TSC) (3, 4). The latter is due to an autosomal dominant mutation of the TSC1 (9q34) or TSC2 (16q13.3) genes, with activation of the mTOR intracellular signaling pathway, associated with a multisystemic disease, a greater number of lesions, a higher growth rate (1.25 cm/year), lower mean age at diagnosis (18 years), and considerable complications during follow-up (4, 8–10).

With regard to its histological classification, there are two subtypes of renal AML, classic and epithelioid. The classic subtype has been characterized in the active surveillance (AS) series, documenting slow growth and a low rate of complications in sporadic cases (2, 11). The epithelioid subtype encompasses 3.9% of renal AML, classified in 2004 by the World Health Organization as a potentially malignant neoplasm with aggressive behavior, and one-third of cases showing local invasion and metastasis at the time of diagnosis (3, 4, 12).

The clinical presentation of renal AML is generally asymptomatic; in 80% of the cases, it is found incidentally in diagnostic images (1, 13–15). Despite this, a classic triad of abdominal pain, palpable mass, and hematuria is described in 40% of the cases (4, 16). The diagnosis is made by the presence of macroscopic fat on images, mainly noncontrast computed tomography (CT) (1, 9).

The main complications of renal AML are chronic kidney disease (CKD), with a rate five times higher than the general population, and spontaneous retroperitoneal hemorrhage, present in 10–15% of patients, which may cause hypovolemic shock in up to 30% of these patients (1, 6, 17). The risk of bleeding is associated with the size of the lesion, aneurysms >5 mm, gravidity, TSC, anticoagulation, and trauma (1). Aggressive behavior and concomitant malignancy are also important but less prevalent complications (6).

Currently, the main therapeutic objective is to avoid complications, while preserving renal function. Indications for active treatment include growth during follow-up, associated symptoms, suspicion of malignancy, bleeding (hematuria or retroperitoneal hemorrhage), and size. This last indication has generated considerable controversy in the most recent scientific publications, which suggest that it is not an isolated predictor of complications, and that the symptoms and other imaging parameters should be given more importance. Recent studies in AS have modeled the natural history of AML, documenting a slow growth rate, low risk of surgical complications, and overtreatment for lesions >4 cm in diameter (4, 11, 18).

Current therapeutic options for preventive and active treatment are AS, selective arterial embolization (SAE), ablation therapies, surgical management, and mTOR inhibitors in patients with TSC-associated AML (3, 8, 19).

The purpose of this article is to perform an updated literature review on the diagnosis, therapeutic options, and criteria for invasive intervention in patients with renal AML.

## Methods

The primary search protocol was performed using the PubMed, Embase, and LILACS databases using the MeSH terms “angiomyolipoma,” “kidney,” “kidney neoplasm,” “diagnosis,” and “therapeutics.” The search was limited to studies in adults aged 18 years or older published within the last 20 years, written in English or Spanish. Articles were included if they reported on the diagnosis and treatment options for sporadic, TSC-associated, or epithelioid AML. All titles and abstracts were assessed by two of the authors and included according to their contribution to the objective of the article. Some additional references were incorporated given their clinical and historical relevance. We excluded from the primary protocol all duplicated registries, editorial letters, and articles concerning pediatric population.

## Results

**Figure 1:**
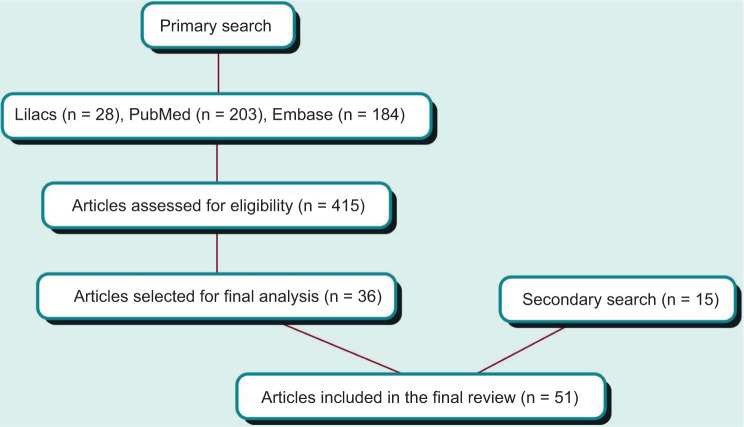
Literature review algorithm.

A total of 415 articles were found after the primary search protocol. According to the inclusion criteria, we included 36 articles for the final analysis, while another 15 articles were included by consensus based on their historical and clinical relevance (Figure 1).

## Discussion

### Diagnosis

The diagnostic approximation of classical AML by noncontrast CT is based on the presence of fat in a renal mass, defined in Hounsfield units (HU) as −10 (−15 to −30 HU) (4). Likewise, renal AML can be classified as fat-rich, fat-poor, and fat-invisible using CT or MRI (20). It should be kept in mind that this finding can also be seen in renal cell carcinoma (RCC), lipoma, liposarcoma, oncocytoma, Wilms tumor, and teratoma; therefore, other characteristics such as the contrast enhancement pattern and the presence of calcifications must also be considered (3, 4, 21). Some research centers have reported cases of AML concomitant with renal cancer in 1% of the patients (6).

In search of a more accurate imaging diagnosis, tomographic techniques such as pixel mapping have emerged, improving the specificity for fat detection by up to 100%, along with advanced magnetic resonance techniques, such as chemical shift, FLASH, India Ink, gradient echo, and fat suppression (9). Within a retrospective study, it was documented that 81.4% of sporadic AML cases, 47.1% of those associated with TSC, and 78% of epithelioid AMLs were diagnosed with ultrasound (US) and tomography. Less than 8% of the cases (1% for sporadic AML and 7.8% for TSC-associated AML) were diagnosed using MRI, with the exception of the epithelioid subtype (21.^7^%) (7).

After the suspicion of AML in imaging, a definitive diagnosis must be made by histopathology, highlighting the importance of the pathologist and the utility of preoperative diagnostic percutaneous biopsy in inconclusive or complex cases (14, 19, 22). Percutaneous biopsy histology is concordant with final pathology in 93% of the cases with a series reporting a complication rate of only 1.5% (23).

### Sporadic AML

For the management of AML, the following have to be considered: symptoms (intractable pain, hematuria), suspicion of malignancy, the risk of retroperitoneal hemorrhage (women of childbearing age, size), growth during follow-up (>0.25 cm/year), inadequate access to an emergency department, and episodes of spontaneous rupture (1, 5, 14, 24, 25).

The most widespread cutoff point for treatment has been a diameter of >4 cm. This imaging finding is present in 82–94% of symptomatic patients, without being a sine qua non, with other series reporting symptoms in only 30% of the cases (9, 14). Using 4 cm as a cutoff value to predict retroperitoneal bleeding is very sensitive (100%) but not very specific (38%) and yields many false positives (4). Ouzaid’s study in AS concluded that treatment based on a cutoff size of ≥4 cm caused overtreatment in 65% of the patients, and identified an increased risk of bleeding in lesions of >6 cm in diameter (11). In concordance with these findings, Kuusk et al. found an association with bleeding in 74%, 17%, and 9%, related to sizes of >6 cm, 4–6 cm, and <4 cm, respectively (1).

In a review of statistically significant publications, it was identified that the risk of bleeding is greater for lesions with a mean diameter of 8 cm versus 4 cm (P < 0.001) (7). This review included the study by Yamakado et al. where hemorrhage occurred in lesions with an average size of 11.4 cm versus 5 cm in nonhemorrhagic lesions. Finally, the authors identified a cutoff size of ≥7.35 cm as the best predictor of bleeding, with this finding present in 36% of the bleeding patients analyzed (26).

Gandhi et al. published their experience with CT angiography in AML, where they evaluated predictors of rupture. Using a cutoff size of >4 cm had a sensitivity of 20%, specificity of 89%, positive predictive value (PPV) of 83.3%, and a negative predictive value (NPV) of 28.5%, while the presence of an aneurysm of >5 mm had a sensitivity of 75%, specificity of 90%, PPV of 50%, and NPV of 96.4% (27). Other studies have shown an association between the presence of aneurysmal vessels with a diameter of >5 mm and an increased risk of retroperitoneal bleeding, with a sensitivity of 100% and a specificity of 86% (26, 28). Likewise, other studies including a review by Murray et al. identified that high vascularity and the presence of tortuous vessels were potential risk factors for retroperitoneal bleeding (9, 29, 30).

Other risk factors for bleeding are accelerated growth, association with TSC, and pregnancy status (6, 11, 17). Concerning AML during pregnancy, no clinical studies or large case series are available; case reports suggest a hormonal effect that conditions an increased growth rate (31). An elevated risk of rupture and obstetric complications have been related to vaginal delivery with a possible benefit in scheduling cesarean delivery (6, 18). Raft et al. reported 72 cases of AML in pregnancy, 58 of which experienced rupture at an average gestational age of 27 weeks, 30% presented hemorrhagic shock, and 13% were associated with fetal death (32). Likewise, there have been case reports where active management was necessary in pregnant women (nephrectomy, embolization) (6).

### TSC-Associated AML

Angiomyolipoma associated with TSC usually has systemic manifestations (central nervous system, heart, lungs, and skin). The mutation in the TSC1 and TSC2 genes conditions the activation of the mTOR-PI3K/AKT pathway, with renal compromise as the most common cause of death in adults (14, 17, 33). Renal AML is present in up to 80% of the patients with TSC. Patients are predominantly young (mean age of 18 years), with a tendency for rapid growth (0.55–1.25 cm/year), more symptoms, larger lesions (mean 3.5–19.3 cm), 90.2% bilateral and 94.1% multiple in their presentation, without a sex difference in incidence (7, 17). In addition to the increased risk for retroperitoneal hemorrhage, complications of TSC-associated renal AML are chronic arterial hypertension and CKD, the latter with a fivefold higher rate and a 30-year earlier onset (CKD stage 3) compared to the general population (3, 8, 17).

Management with mTOR inhibitors currently has evidence in TSC-associated AML and LAM, without being able to document its usefulness in sporadic cases. Everolimus has more robust evidence and is currently the only FDA-approved drug for TSC-associated renal and LAM. This is indicated by the Tuberous Sclerosis Renal Guidelines with an effectiveness against placebo demonstrated by the EXIST-1 and EXIST-2 trials, indicating a reduction of 50% in size in lesions of >3 cm in 42–54% of the patients. Likewise, a sustained volume reduction of up to 192 weeks, no bleeding, and a statistically significant lower rate of progression was observed in the high-risk population. Its main adverse effects include stomatitis, nasopharyngitis, acne, proteinuria, headache, cough, and hypercholesterolemia (3, 17, 34, 35).

### Epithelioid AML

According to the current literature, it represents approximately 3.9% of the renal AML cases and ≤1% of the renal tumors (6, 7, 12). It appears predominantly in women (3:1 ratio); up to 79% are symptomatic, with a mean age at diagnosis of 46 years, a mean size of 10.5 cm, and a more aggressive behavior, with an overall survival rate of 50% at 3 years, and one-third of the patients with local extension or metastasis at diagnosis (6, 9). Its imaging diagnosis is more complex because these are tumors with a lower fat content, in many cases with aggressive characteristics (venous extension, distant metastases), making it harder to differentiate from RCC, with a requirement for MRI or histopathology in 21.7% and 7.7% of cases, respectively, to clarify the diagnosis (6, 7).

The prognostic factors identified for aggressive behavior include lesion size of >7 cm, extrarenal extension, young age, tumor necrosis, and an epithelioid histological pattern (3, 36). This subtype is treated as RCC, given its risk of aggressive behavior and high recurrence rate (9).

### Choosing a treatment option

Understanding the natural history of renal AML has changed the therapeutic paradigm, with a current trend toward a more conservative approach. Active surveillance is the first-line intervention in most cases. It seeks to identify low-risk renal AML cases, qualified for close follow-up, and supervise early indicators of complications, mainly rupture or retroperitoneal bleeding, to offer timely treatment.

In the Ouzaid et al. series, 130 patients underwent AS, 17 (13%) required active treatment at the mean follow-up of 49 months, three patients (2.3%) due to retroperitoneal hemorrhage. In a univariate analysis, predictors of late intervention included a larger tumor size (>4 cm), a higher body mass index, contralateral lesions, and symptomatic disease (11). Despite associating a 4 cm cutoff size as a predictor for intervention, it was evident that it led to overtreatment. It was found that 67% of the symptomatic patients managed with AS did not require subsequent intervention, making necessary an assessment of symptomatic severity and the possibility of conservative management (1, 11).

Bhatt’s retrospective series evaluated growth in cases of sporadic AML without treatment, finding that >92% of the asymptomatic or oligosymptomatic cases do not grow or grow very slowly regardless of their initial size (> or < 4 cm) in a follow-up at 43 months (37). Therefore, they recommend AS in sporadic AML regardless of size in asymptomatic patients, evaluating treatment if a rapid growth rate (>0.25 cm/year) is present during follow-up (24, 37). In a systematic review from 2015, 44 studies with 2,580 patients were included, 281 presented spontaneous rupture with only five deaths (1.9%); all deaths in this series were related to TSC (18, 29). A different series of AS documented only 2.2% of patients with hematuria or retroperitoneal bleeding during follow-up, with 5.7% requiring active treatment (19).

Currently, there is no guideline that standardizes the frequency of follow-up. The existing protocols are based on AS studies that suggest physical examination, renal function, and CT or US at 6 and 12 months, and then annually, with closer follow-up intervals in high-risk populations (2, 9, 14, 19). With regard to TSC-associated AML, the 2012 Tuberous Sclerosis Renal Guidelines consensus suggests MRI follow-up every 1–3 years (17).

Selective arterial embolization has become the first line of management, being a safe, minimally invasive alternative, with a success rate of 90–100%, and emergency control in 96–100% of the patients (6, 9). It is useful in symptomatic patients, in prophylactic and presurgical management, and acute retroperitoneal hemorrhage (38). The embolic agents used are ethanol, polyvinyl alcohol, gelfoam, coils, and triacyl gelatin microspheres, without studies documenting superiority of any over the others (39). In the study by Kothary et al., the use of an ethanol-based agent over polyvinyl alcohol was suggested, without recommending the use of coils due to the risk of collateral formation (40, 41). It has a grade C indication in prophylactic management of hemorrhage in growing AML of > 0.25cm/year, and/or the presence of aneurysms of >5 mm. There are no studies comparing prophylactic embolization versus mTOR inhibitors in the TSC-associated AML population (24). The recurrence rate in the results of 16 series is 25%, with a reintervention requirement in up to 37% of the patients managed with this technique (11, 14, 17). The role of SAE prior to partial nephrectomy reduces, according to Tan et al. blood loss, hospital stay, and residual tumor in renal AML cases of >7 cm (42). In addition, 93.3% of the patients who received preoperative SAE preserved their kidneys versus 33.3% who did not receive prior SAE and later required radical nephrectomy intraoperatively (8).

In prophylactic and symptomatic management, SAE offers a 43% reduction in the volume of renal AML, with other studies reporting a 26–99% decrease in the volume of lesions (11, 42). Among its advantages, it conditions fewer major complications compared to surgery, less bleeding, and a shorter hospital stay (5). Its most prevalent complication is post-embolization syndrome (PES), present in up to 89% of the cases, characterized by fever, flank pain, and leukocytosis; it improves with symptomatic management (3). The study by Bissler et al. evidenced a 30% decrease in PES with the use of corticosteroids and a prophylactic antibiotic, with the need for more studies to be recommended in clinical practice (9, 43). Other described complications are vascular injury, abscess formation, pleural effusion, renal infarction, and impact in glomerular filtration rate (GFR) (2). A series of 45 AML cases taken to SAE did not manifest any bleeding, nor did they require renal replacement therapy; there were also no deaths in the following 14 months (42). Another series of 71 patients over 10 years reported a complete loss of renal unit function in 2.9% of the patients (44). Arguments against this technique include a study in which AML cases of ≥8 cm had a hazard ratio of 2.36 (P < 0.05) for reintervention (1).

Ablation arises as an alternative for the management of small and asymptomatic renal AML (<4 cm), with few studies, and without high-level evidence, but with promising results. Ablation has been considered superior to Nephron-sparing surgery (NSS) in comparative studies with regard to renal function preservation (45). The most studied technique has been radiofrequency ablation with two series that have documented good effectiveness, low reintervention rates, and minor complications during follow-up (19). Prevoo et al. reported the successful case of a sporadic 4.5 cm renal AML managed with radiofrequency ablation in a solitary kidney without recurrence as evidenced in imaging, and with preservation of renal function at 12 months (46). Evidence on the clinical utility of this technique, along with cryoablation and microwave ablation, is still lacking (2, 3).

Surgical management has been displaced to a second plane with the advent of SAE, with the advantage of NSS over radical nephrectomy (RN), due to the already well-understood relationship with CKD and increased associated morbidity and mortality (3). Radical nephrectomy conditions twice the GFR compromise compared to NSS in the immediate postoperative period; it is only indicated in AML rupture with retroperitoneal bleeding and uncontrolled hypovolemic shock after failed embolization (4, 47).

Boorjian et al. described in the largest series of sporadic AML and open NSS, at 8 years of follow-up, a recurrence rate of 3.4%, and 12% de novo CKD, respectively (48). Berglund et al. reported a 14% loss of renal function due to a requirement for conversion to RN (49). In addition, Minervini et al. revealed less blood loss, shorter ischemic time, and hospital stay with NSS when compared with RN. Nephron-sparing surgery presents surgical complications of 21.4% with a very low reintervention requirement (<1%) (6, 19). Additional series report an 86.9% preservation of the GFR, without complications or recurrence (6). Despite new trends, a systematic review of the European Association of Urology in 2019 concluded that NSS had similar morbidity to SAE but seemed to be the most effective option to prevent recurrence and the need for secondary treatments (19).

Table 1 summarizes some of the most important series in terms of different treatment modalities, their success rate, and main points to consider.

**Table 1:** Case series in renal AML management.

Study	Treatment modality	Success rate/Response	Effects/Adverse events
EXIST 1 (17)	Everolimus in TSC-AML	53.3%	Stomatitis, nasopharyngitis, headache, acne, hyperlipidemia, hematologic disorders
EXIST 2 (17)	Everolimus in TSC-AML	58%	Stomatitis, nasopharyngitis, headache, acne, hyperlipidemia, hematologic disorders
Ouzaid et al. (11)	Active surveillance	87%*	Active treatment in 13% of the patients during follow-up
Bardin et al. (50)	SAE	96%	Recurrence in 13%, repeat embolization in 17%, PES in 80%
Murray et al. (29)	SAE	93.3%	PES in 35.9%, repeat embolization in 20.9%
Lin et al. (51)	Robotic-assisted NSS	100%	CKD in 10%, perioperative complications Clavien ≤ II in 26%
Castle et al. (45)	Radiofrequency ablation in sporadic AML	100%	Perioperative complications in 13.3%
Kuusk et al. (1)	Radical nephrectomy	100%	Bleeding in 10.8%, no reinterventions
Boorjian et al. (48)	NSS	96.6%	Complications in 12%, no CKD

TSC-AML – TSC-associated angiomyolipoma; SAE – selective arterial embolization; PES – post-embolization syndrome; NSS – nephron-sparing surgery; CKD – chronic kidney disease.

* Patients did not require active treatment during follow-up.

### Considerations on available evidence and management proposal

Research in renal AML has led us to understand this renal tumor as a heterogeneous pathology, with a variable natural history needing different therapeutic strategies. The mainstay in management is symptomatic control and prevention of morbidity and mortality, with a specific focus on retroperitoneal bleeding and secondary hemorrhagic shock.

Active surveillance as expectant therapy has permitted the study of the natural history of AML, managing to identify early indicators of treatment. The conservative management approach is based on a slow growth rate, the risks of overtreatment, the low risk of rupture, and the related mortality.

As for symptomatic patients, TSC-associated AML, pregnant women or those of childbearing age, aneurysms of >5 mm, and those with rapid growth rates, represent a high-risk population qualifying for early interventions. Likewise, among the interventions, minimally invasive techniques predominate as the first line of treatment, with the emergence of new thermal ablation therapies with promising results.

**Figure 2:**
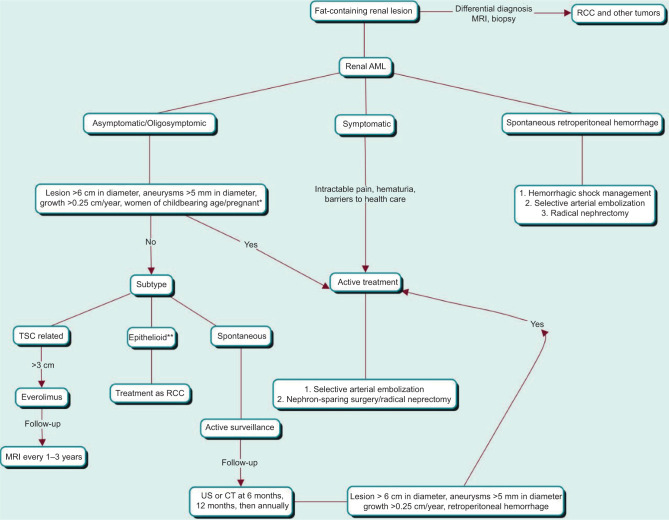
Proposed updated management algorithm. RCC – renal cell carcinoma; AML – angiomyolipoma; TSC – tuberous sclerosis complex; MRI – magnetic resonance imaging; US – ultrasound; CT – computerized tomography. *Associated with other risk factors, according to medical criteria. **See characteristics and risk factors in “epithelioid AML.”

Undoubtedly, studies and guidelines that standardize the management of renal AML are lacking in order to facilitate a focused approach in the urological community. Figure 2 outlines a proposed management algorithm based on current evidence.

## Future perspective

Due to the low incidence of renal AML, there is a scarcity of high-level evidence comparing the various treatment options for this pathology. In terms of future research, there is a necessity for updated clinical guidelines that assist the physician toward suggesting a treatment that minimizes morbidity and mortality. Furthermore, translational research in immunotherapy or gene therapy might be the key to reaching a potential cure in the future.

## Conclusion

Renal AML is a pathology of urological management with low prevalence but with a significant risk of morbidity and mortality. Recent data have allowed us to define CT as the ideal diagnostic method and postulate that patients with growth >0.25 cm/year, intralesional aneurysms of >5 mm, uncomfortable symptoms, pregnancy status, and women of childbearing age are the ideal candidates for active management.

Despite the limitations of the available evidence, it is possible to consider AS, NSS, and the use of mTOR inhibitors as management alternatives for selected patients.
